# Zika Virus Infection Results in Biochemical Changes Associated With RNA Editing, Inflammatory and Antiviral Responses in *Aedes albopictus*

**DOI:** 10.3389/fmicb.2020.559035

**Published:** 2020-10-02

**Authors:** Maria G. Onyango, Geoffrey M. Attardo, Erin Taylor Kelly, Sean M. Bialosuknia, Jessica Stout, Elyse Banker, Lili Kuo, Alexander T. Ciota, Laura D. Kramer

**Affiliations:** ^1^Wadsworth Center, New York State Department of Health, Slingerlands, NY, United States; ^2^Department of Entomology and Nematology, University of California, Davis, Davis, CA, United States; ^3^School of Public Health, State University of New York, Albany, NY, United States

**Keywords:** Zika virus, *Aedes albopictus*, primary metabolites, lipids, biogenic amines, metabolomic phenotyping

## Abstract

Rapid and significant range expansion of both the Zika virus (ZIKV) and its *Aedes* vector species has resulted in the declaration of ZIKV as a global health threat. Successful transmission of ZIKV by its vector requires a complex series of interactions between these entities including the establishment, replication and dissemination of the virus within the mosquito. The metabolic conditions within the mosquito tissues play a critical role in mediating the crucial processes of viral infection and replication and represent targets for prevention of virus transmission. In this study, we carried out a comprehensive metabolomic phenotyping of ZIKV infected and uninfected *Ae. albopictus* by untargeted analysis of primary metabolites, lipids and biogenic amines. We performed a comparative metabolomic study of infection state with the aim of understanding the biochemical changes resulting from the interaction between the ZIKV and its vector. We have demonstrated that ZIKV infection results in changes to the cellular metabolic environment including a significant enrichment of inosine and pseudo-uridine (Ψ) levels which may be associated with RNA editing activity. In addition, infected mosquitoes demonstrate a hypoglycemic phenotype and show significant increases in the abundance of metabolites such as prostaglandin H2, leukotriene D4 and protoporphyrinogen IX which are associated with antiviral activity. These provide a basis for understanding the biochemical response to ZIKV infection and pathology in the vector. Future mechanistic studies targeting these ZIKV infection responsive metabolites and their associated biosynthetic pathways can provide inroads to identification of mosquito antiviral responses with infection blocking potential.

## Introduction

Zika virus (ZIKV) was first isolated in Zika forest, Uganda from a sentinel rhesus monkey in 1947 as well as from *Aedes* (Stegomyia) *africana* (Dick, 1952; Dick et al., 1952; Hayes, 2009; Petersen et al., 2016) and is a member of the family Flaviviridae, genus *Flavivirus* with a 10,794 base positive-sense single stranded RNA genome (Faye et al., 2014), classified as a member of the Spondweni group (Cook and Holmes, 2006). The virus is primarily transmitted by mosquitoes of the genus *Aedes* (Wikan and Smith, 2016). Recently, ZIKV has become a focus of intense research due to its rapid geographic spread in the Americas as well as its association with birth defects in offspring from infected mothers (e.g., microcephaly) and neurological syndromes (Ventura et al., 2016; de Oliveira et al., 2017; World Health Organization [WHO], 2019). This has resulted in a concerted effort to understand the biology of ZIKV and the interactions it has with its vector host.

The main route of ZIKV transmission is by the bite of infected *Aedes* mosquitoes, in addition, sexual, transplacental, and blood transfusion have been documented (Petersen et al., 2016). At the moment, no FDA-approved medication or vaccine to treat or prevent ZIKV infection exists (Gulland, 2016). Symptomatic relief by medications are the only source of relief for infected individuals (Kazmi et al., 2020). Zika disease control is achieved primarily by prevention of mosquito bites, vector abatement and prevention of sexual transmission (Petersen et al., 2016). Despite the progress made in understanding Zika etiology and its transmission, there exists a significant knowledge gap in the understanding of interactions between ZIKV and its vector.

The biochemical interactions and chemical processes within a living organism is collectively known as its metabolism. Metabolites are the basic units of cellular function, involved in enzyme-catalyzed chemical processes and are important for cellular function (Schrompe-Rutledge et al., 2016). The cascade of biological information in a living organism flows from the genes, to the transcripts through proteins and subsequently to the metabolites. Hence, any perturbations observed at the metabolome level is a direct consequence of alterations in the genome, transcriptome and the proteome of a biological system. In addition, the metabolome of an organism is considered a reflection of the chemical phenotype of that organism. In fact, metabolomics is considered a promising link between the genotype and phenotype gap (Bujak et al., 2015). Because of the sensitive nature of metabolomics, any alterations in the biological pathways can result in the understanding of the mechanisms underlying physiological conditions and processes resulting from disease progression in an organism (Johnson et al., 2016).

The study of metabolomics perturbations due to viral infection is very important due to the potential identification of metabolites that distinguish disease states and outcomes. The identification of these metabolites could lead to diagnostic and therapeutic targets. In addition, it facilitates a deeper understanding of the vector pathogen interactions (Byers et al., 2019).

Using high-resolution mass spectrometry of mosquito cells, Perera et al. (2012) demonstrated that DENV infection resulted in enrichment of lipids that modulates the curvature of membranes as well as those which influence the permeability of membranes. Further, the same study showed an increase in sphingolipids and other bioactive signaling molecules involved in control of membrane fusion, fission and trafficking. In addition, molecules associated with cytoskeletal reorganization were enriched in DENV infected cells. Melo et al. (2016), utilized a lipidomics approach on ZIKV-infected C6/36 mosquito cell lines in a bid to characterize new biomarkers of ZIKV infection as well as establish new targets for viral controls in vertebrates and invertebrate vectors. They identified 13 lipids as specific marker for ZIKV infection. Those lipids were associated with intracellular mechanism of viral replication and or cell recognition. These findings are similar to that of Chotiwan et al. (2018). They analyzed the temporal metabolic perturbations associated with DENV infection of the midgut tissue in *Ae. aegypti* and observed an increase in glycerophospholipids, sphingolipids and fatty acyls that accompanied DENV replication. Levels of acyl-carnitines were also enriched while points in the sphingolipid pathway influencing dihydroceramide to ceramide ratios were noted as very important for the life of the virus in that study.

Furthermore, Bottino-Rojas et al. (2019) utilized a whole cell lipidomics approach to interrogate lipid perturbations in West Nile virus strain Kunjin (WNV_KUN_) infected Vero cells. This reveal elevated levels of phospholipase A2 activity in the infected cells resulting in increased production of lyso-phosphatidylcholine moieties associated with subcellular sites of viral replication.

To understand the role of oxidative stress in combating arboviral infections in mosquitoes, Shrinet et al. (2018) carried out a metabolomics analysis of *Aedes* mosquitoes infected and co-infected with Chikungunya (CHIKV) and DENV. Further, they obtained different -omics data from the public database including proteomics and transcriptomics. The data analysis demonstrated that the different pathways were harmonized in their response to infection in regulating oxidative stress during arboviral infection of *Aedes* mosquitoes.

The goal of this study is to provide a comprehensive analysis of dynamic changes in the global metabolic profile (primary metabolites, lipids and biogenic amines) in whole *Ae. albopictus* mosquitoes infected with ZIKV. We identified an array of metabolic biomarkers that change in abundance in response to ZIKV infection. These biomarkers provide insights into how the virus interacts with its vector and help to elucidate potential novel barriers to transmission.

## Materials and Methods

### Mosquitoes Species and Maintenance

All experimental protocols in this study were approved by Wadsworth Institutional Review Board. *Aedes albopictus* were collected from Suffolk County, NYS in 2015 (kindly provided by I. Rochlin) and colonized at the New York State Department of Health (NYSDOH) Arbovirus Laboratory. The mosquito strain was continually maintained at 28°C with relative humidity of 60% and 16:8 h light: dark (LD) photoperiod under standard rearing conditions. To confirm the temperature and humidity of the chambers, both internal chamber log and independent HOBO data loggers (Onset, Cape Cod, MA, United States) were used. F15 *Ae. albopictus* were vacuum-hatched and the immature stages reared under the standard rearing conditions in the lab. The larvae were fed on Tetra Pond Koi growth feed, the adults were transferred to 3.8 L cardboard cartons and allowed to mate for 5 days while being provided with sugar and water *ad libitum*. Water and sugar were withdrawn 24 h before being offered 8.3 log_10_ PFU/ml ZIKV HND (Ciota et al., 2017) blood meal. The control group were provided a non-infectious blood meal.

### Blood Meal Preparations and Mosquito Viral Infections

We used frozen ZIKV HND (2016-19563, GenBank accession no. KX906952) strain. The infectious blood meal consisted of 1:1 dilution of defibrinated sheep blood plus 2.5% sucrose; sodium bicarbonate (to adjust pH to 8.0) and virus. Infectious blood meals contained a viral titer of 8.3 log_10_ PFU/ml ZIKV HND (Ciota et al., 2017; Onyango et al., 2020). The non-infectious blood meal (negative control) contained a final concentration of 2.5% sucrose solution. The female mosquitoes were exposed to blood meals in a 37°C pre-heated Hemotek membrane feeding system (Discovery Workshops, Acrington, United Kingdom) with a porcine sausage casing membrane. All methods were carried out in accordance with relevant guidelines and regulations. Both blood meals were offered to the female mosquitoes after withdrawal of sugar 24 h before blood feeding. After an hour, the mosquitoes were anesthetized with CO_2_ and immobilized on a pre-chilled tray connected to 100% CO_2__._ Engorged females were separated and placed in three separate 0.6 L cardboard cartons (30 individuals per carton). In addition, 1 ml of each blood meal was transferred to a 1.5 ml Safe Seal microtube (Eppendorf, Hamburg Germany) and stored at −80°C. Blood fed females were maintained on 10% sucrose solution provided *ad libitum*.

### Sample Collection for Metabolomics Analysis

#### Mosquito Dissection

To reduce the amount of concentrated carbohydrates in the mosquitoes in order to avoid disruption to the mass spectrometer, sucrose solution was withdrawn from the mosquito cages at 4 days post infection (dpi) and replaced with plain water. At 7 dpi, the female mosquitoes were immobilized using triethylamine (Sigma Aldrich, St. Louis, MO, United States). To examine for ZIKV dissemination in the mosquito, the legs were removed from the mosquitoes and placed in individual tubes containing 500 μl mosquito diluent (20% heat-inactivated fetal bovine serum in Dulbecco phosphate-buffered saline plus 50 μg/ml penicillin/streptomycin, 50 μg/ml gentamicin and 2 μg/ml Fungizone [Sigma Aldrich, St. Louis, MO, United States]) and a bead. All samples were held at −80°C until assayed. The corresponding whole bodies were snap frozen and stored at −80°C. To ensure uniformity during metabolite extraction, the legs of the individual female mosquitoes exposed to non-infectious blood meal were also removed and the whole bodies snap frozen and stored at −80°C.

### Quantitative RT-PCR Screening of ZIKV in *Ae. albopictus*

A ZIKV-specific quantitative PCR assay that targets the NS1 region was utilized to obtain viral titer (Lanciotti et al., 1992) from the legs as described by Ciota et al. (2017). Individual female mosquitoes showing a disseminated virus infection (ZIKV-positive legs) were identified as “ZIKV-infected mosquitoes.” A threshold C_t_ value of 35 was used to discriminate disseminated from non-disseminated individuals. In order to perform metabolite extraction, both the individuals that were exposed infectious (and were disseminating ZIKV) as well as those exposed to the non-infectious blood meal were pooled. The pools consisted of five individuals and ten biological replicates per sample types. The samples were frozen at −80°C before submission to the NIH West Coast Metabolomics Center at UC Davis. Microfuge tubes handled by the different individuals involved in the sample preparations were also sent to the core for normalization of the variation introduced during sample collection and preparation.

To characterize the metabolic changes incurred by *Ae. albopictus* during ZIKV infection, we performed metabolites analysis on whole bodies of *Ae. albopictus* 7 dpi with ZIKV and analyzed primary metabolites, lipids and biogenic amines.

### Metabolite Extraction

A total of 20 pools (*N* = 10 replicates per infection status; *n* = 5 mosquitoes per pool) of whole body *Ae. albopictus* females exposed to infectious and non-infectious ZIKV blood meals were analyzed using three untargeted metabolomic panels to identify metabolic perturbations associated with ZIKV infection. The three analyses utilized were designed to screen for relative differences in the abundance of primary metabolites, lipids and biogenic amines. Each replicate was partitioned into three fractions using the protocol described in Matyash et al. (2008). Briefly, each of the experimental treatments were homogenized and vortexed with a bead mill homogenizer (FastPrepa-24 MP Biomedicals) in 975 μl of ice-cold methanol: methyl-tert-butyl ether (3:10) extraction solution. Phase separation of the homogenate was induced by the ether (3:10) extraction solution. Phase separation of the homogenate was induced by the addition of 188 μl of LC-MS grade water followed by 6 min of shaking at 4°C. tubes were then vortexed for 20 s and then centrifuged for 2 min at 14,000 × *g*. The organic phase was transferred to a separate tube for lipidomics analysis. The remaining aqueous fraction was split into two aliquots for the primary metabolite and biogenic amine analysis panels. Quality control samples were generated for each of the three metabolite panels by pooling 100 μl of each replicate from both experimental treatments. Blank controls consisting of empty tubes were also included in the analysis to account for contaminants associated with handling. The aqueous phase aliquots were dried down by centrifugation under vacuum in a speedvac.

### Primary Metabolite Analysis by GC TOF

Primary metabolites (including sugars, amino acids, hydroxyl acids and related biochemical pathways) were profiled using the Gas Chromatography time of flight (GC TOF) untargeted protocol as described in Fiehn (2017). Dried sample aliquots were resuspended in 450 μl of nitrogen-degassed 50:50 (v/v) acetonitrile/water at room temperature. Resuspended samples were centrifuged for 2 min at 14,000 × *g* at room temperature. Supernatant was transferred to anew tube and dried down in a speedvac evaporator.

Samples and controls were derivatized (chemical conversion of non-volatile compounds to volatile compounds) by treatment of dried extracts with 10 μl of 20 mg/ml methoxamine hydrochloride solution in pyridine. Samples were then shaken at maximum speed on an orbital heating plate for 1.5 h at 30°C. Then 91 μl of *N*-methyl-*N*-(trimethylsilyl)-trifluoroacetamide (MSTFA) was added to each sample and controls which were shaken at maximum speed for 30 min at 37°C. Derivatized samples and controls were then added to glass vials for GC-MS analysis and data acquisition.

Samples were injected via an Agilent 7693 autosampler (Santa Clara, CA, United States) into a 7890 Agilent GC fitted with a RtX-5Sil MS (30 m length × 0.25 mm internal diameter with 0.25 μm film made of 95% dimethyl/5% diphenylpolysiloxane) Restek corporation (Middelburg, ZEELAND, Netherlands) chromatographic column using helium as a mobile phase at 1 ml/min. Sample volumes of 0.5 μl were injected at 50°C ramped to 250°C by 12°C/s. the oven temperature program was 50°C for 1 min then ramped at 20°C/min to 330°C, held constant for 5 min.

Data acquisition was performed with a Leco Pegasus IV time-of-flight MS instrument (Leco, St. Joseph/MI, United States). Mass spectrometry parameters were used as follows: the mass spectrometer is run with unit mass resolution at 17 spectra/s from 80 to 500 Da at 70 eV ionization energy and 1800 V detector voltage with a 230°C transfer line and a 250°C ion source. The multi baffled glass injection liners were changed every ten injections followed by reinjection of the pooled quality control sample throughout the course of the analytical batch.

### Lipidomic Profiling by LC/Q-TOF CSH

Lipidomic analysis was performed by liquid chromatography (LC) coupled to a quadruple time-of flight (QTOF) charged surface hybrid column (CSH) mass spectrometer (Waters MS Technologies, Manchester, United Kingdom). The lipid containing organic phase from the metabolite extractions (350 μl) were dried in a Speed Vac (Thermo Fisher, Pittsburgh, United States). The remaining residue was redissolved in 110 μl MeOH:Tol (9:1) including an internal standard mix (50 ng/ml) (see Supplementary Table 2 for lipidomic internal standard composition). The resuspended samples were then vortexed for 10 s, sonicated for 5 min and centrifuged for 2 min at 16,100 × *g*. The supernatant was aliquoted into two tubes for analysis in positive and negative electrospray ionization modes.

The data was analyzed on an Agilent 6530 Q-TOF LC/MS UPLC mass spectrometer. Samples were separated on a C_18_ column equipped with charged surface hybrid column.

Data acquisition was attained as described in Dong et al. (2017). Briefly, each sample was injected at 4°C into a Waters Acquity UPLC CSH C_18_ column (100 mm length × 2.1 mm internal diameter; 1.7 μm particles) at 65°C with the flow rate set at 6 ml/min.

Each sample was subjected to electrospray ionization (ESI) in positive and negative modes with a capillary voltage of 3.2 kV, cone voltage of 30 kV and desolvation gas temperature of 400°C. positive and negative specific ionization settings are as follows:

Positive mode settings:Spectrophotometer: Agilent 6530 Q-TOF LC/MS UPLCColumn: Waters Acquity UPLC CSH C18 (100 mm length × 2.1 mm internal diameter; 1.7 μm particles)Mobile phase (A): 60:40 v/v acetonitrile:water + 10 mM ammonium formate + 0.1% formic acidMobile phase (B): 90:10 v/v isopropanol:acetonitrile + 10 mM ammonium formate + 0.1% formic acidScan range positive mode: m/z 120- 1200 DaInjection volume: 1.7 μlESI capillary voltage: ESI (+): + 3.5 kV

Negative mode settings:Spectrophotometer: Agilent 6530 Q-TOF LC/MS UPLCColumn: Waters Acquity UPLC CSH C18 (100 mm length × 2.1 mm internal diameter; 1.7 μm particles)Mobile phase (A): 60:40 v/v acetonitrile: water + 10 mM ammonium acetateMobile phase (B): 90:10 v/v isopropanol: acetonitrile + 10 mM ammonium acetateScan range negative mode: m/z 60–1200 DaInjection volume: 5 μlESI capillary voltage: ESI (+): −3.5 kV

The elution gradient for both positive and negative modes was 0 min 15% (B), 0–2 min 30% (B), 2–2.5 MIN 48% (B), 2.5–11 min 82% (B), 11–11.5 min (B), 11.5–12 min 99% (B), 12–12.1 min 15% (B), 12.1–15 min 15% (B).

### Biogenic Amine Analysis Using HILIC LC/Q-TOF

Biogenic amines are low molecular weight organic bases resulting from decarboxylation of free amino acids by animals, plants and microorganisms (Ito and Nakata, 2016). A rapid untargeted analysis of polar metabolites of Zika infected *Ae. albopictus* tissues was carried out by Hydrophilic Interaction Chromatography (HILIC) coupled to Quantum Time of Flight (Q-TOF) mass spectrometry. The analysis was undertaken as described in Chintapalli et al. (2013) with modifications. Dried aliquots for biogenic amine analysis were resuspended in 100 μl acetonitrile (ACN): water (80:20) including internal standards (see Supplementary Table 2 for biogenic amine internal standards) followed by 10 s of vortexing, 15 min of sonication and centrifugation for 2 min at 16,000 × *g*. All processes were performed at 4°C. A pooled quality control sample was run after every 10 samples throughout the course of the analytical batch.

HILIC LC/Q-TOF settings:Spectrophotometer: Agilent 6530 Q-TOF LC/MS UPLCPre-column: Waters Acquity UPLC BEH Amide VanGuard pre-column (1.7 μm, 5 mm × 2.1 mm)Column: Waters Acquity UPLC BEH Amide column, 1.7 μm, 2.1 mm × 150 mm)Mobile phase (A): Solvent A was Ultrapure water with 10 Mm ammonium formiate + 0.125% formic acid, pH 3Mobile phase (B): Solvent B was 95.5 v/v acetonitrile: ultrapure water with 10 mM ammonium formiate + 0.125% formic acid, pH 3Injection volume: 3 μlFlow rate: 400 μl/minESI (+) capillary voltage: + 4.5 kVESI (+) collision energy: + 45 eVScan Range: m/z 60–1200 DaSpectral acquisition speed: 2 spectra/sMass resolution: 10,000

Elution gradient 0 min 100% (B), 0–2 min 100% (B), 2–7 min 70% (B), 7.7–9 min 40% (B), 9.5–10.25 min 30% (B), 10.25–12.75 min 100% (B), 16.75 min 100% (B). The vial tray was kept at a constant temperature of 34°C while the column was set at ambient temperature.

### Data Processing

Data for detected metabolites in all three panels were provided as peak heights for the quantification ion (m/z value) at the associated retention time (rt value). These values were obtained via the following processes for the respective panels.

### Primary Metabolite Panel Data Processing

The raw GC-MS data from the primary metabolite panel was preprocessed in the Leco ChromaTOF version 2.32 software without smoothing using a 3 s peak width baseline subtraction just above the noise level and automatic mass spectral deconvolution/peak detection at signal/noise levels of 5:1. Apex masses were output for comparison against the BinBase metabolomics database (Skogerson et al., 2011). The BinBase algorithm (rtx5) used the following settings: validity of chromatogram (<10 peaks with intensity> 10^7^ counts s^–1^), unbiased retention index marker detection (MS similarity > 800, validity of intensity range for high m/z marker ions), retention index calculation by 5th order polynomial regression. Spectra are cut to 5% base peak abundance and matched to database entries from most to least abundant spectra using the following matching filters: retention index window ± 2000 units (equivalent to about ± 2 s retention time), validation of unique ions and apex masses (unique ion must be included in apexing masses and present at >3% of base peak abundance), mass spectrum similarity must fit criteria dependent on peak purity and signal/noise ratios and a final isomer filter. Failed spectra are automatically entered as new database entries if s/n > 25, purity < 1.0 and presence in the biological study design class was 80%. All thresholds reflect settings for ChromaTOF v 2.32. Quantification is reported as peak height using the unique ion as default, unless a different quantification ion is manually set in the BinBase administration software BinView. A quantification report table is produced for all database entries that are positively detected in more than 10% of the samples of a study design class (as defined in the miniX database) for unidentified metabolites.

### Lipidomics Panel Data Processing

Raw LC/MS spectra were processes with MS-DIAL using the default parameters (Tsugawa et al., 2015). Data from blank samples were used to subtract background and contaminants from sample data based on the max peak height relative to blank average height, the average of all non-zero peak heights for samples, and if the feature is found in at least one sample. This is followed by sample cleanup using MS-FLO to eliminate potential duplicates and isotopes (Defelice et al., 2017). Internal standards served as retention time alignment markers and for quality control purposes. Peaks are annotated in manual comparison of MS/MS spectra and accurate masses of the precursor ion to spectra given in the Fiehn laboratory’s LipidBlast spectral library (Kind et al., 2013). The confidence of annotation was guided by recommendations from the Metabolites Standard Initiative (Sumner et al., 2007). Unique chromatographic features that could not be identified are reported as “unknown.”

### Biogenic Amine Data Processing

The raw data from the biogenic amines panel was processed using mzMine 2.0 (Pluskal et al., 2010) for peak information. Internal standards served as retention time alignment markers and for quality control purposes. Alternatively, selected peaks were collated and constrained into Agilent’s MassHunter quantification method on the accurate mass precursor ion level, using the MS/MS information and the NIST14/Metlin/MassBank libraries to identify metabolites with manual confirmation of adduct ions and spectral scoring accuracy.

### Data Normalization and Analysis

Comparative analysis of the processed data from the ZIKV infected versus uninfected samples was performed using the MetaboAnalyst (V4.0) package (Chong et al., 2018). Raw data were submitted as peak intensity tables with samples in columns in unpaired format. Data were filtered to remove variables with low repeatability using Relative standard deviation (RSD). Samples were normalized by dividing the spectral profile of the sample by the median of all feature intensities of the sample. The data was log transformed and auto scaled (mean-centered and divided by the standard deviation of each variable).

Datasets were analyzed within Metaboanalyst using the following integrated tools and parameters. Significant differences in metabolite abundances between treatment groups were evaluated by Wilcoxon Rank Test assuming unequal variance with a threshold of an adjusted *P*-value (FDR- Benjamini and Hochberg method) of 0.05. Multivariate analysis of the datasets was performed using Partial Least Squares Discriminant Analysis (PLS-DA). Top features from the PLS-DA analyses were signified by associated Variable Importance In Projection (VIP) scores. Heatmaps visualizing relative compound abundances in infected and uninfected samples were generated from top VIP scoring compounds. Distance measures between samples were calculated using Euclidean distance measure algorithm and clustered by the Ward algorithm. Features were standardized by autoscaling.

### Pathway and Network Analysis of Metabolites

Further pathway-based Analysis were performed on the LC-MS data derived from the lipidomic and biogenic amines panel datasets using the Peaks to Pathways function in Metaboanalyst which is a web based iteration of the Mummichog analytical package (Li et al., 2013). Analysis of the primary metabolite dataset via Mummichog was not possible as it was derived from gas chromatography and lacks the required accuracy in mass/charge ratio and retention times required to make accurate metabolite predictions. The pathway analysis placing the experimental observations into relevant biological and disease context. Mummichog utilizes dta from defined metabolic network to predict functional activity directly from feature tables based on mass/charge ratio (mz) and retention time (rt). Data for this analysis were filtered, normalized and scaled using equivalent parameters to those in the section on dataset normalization and analysis. Following filtration and normalization, data were reformatted for submission to the peaks to pathways algorithm. All compounds (including unknowns) were represented by mz, rt, *p*-value. *T*-score and electro spray mode (positive or negative). Reformatted data files representing the lipidomic and biogenic amine panels were combined into a single dataset and submitted for analysis via version 2.0 of the peaks to pathways algorithm using the following parameters: molecular weight tolerance (ppm)- 10, analytical mode- mixed, compounds ranked by *P*-value and primary ions not enforced. Analysis algorithms utilized were Mummichog with a *P*-value cutoff of 0.05 and GSEA using the *Drosophila melanogaster* KEGG library.

Compound identifications resulting from the Peaks to Pathways analysis in the form of KEGG ids were mapped back to annotate unidentified compounds in the original datasets. Compounds with multiple potential annotations were matched with the annotation with the lowest mass difference relative to the compound. Network analysis of KEGG annotated compounds was performed in Cytoscape 3.8.0 (Shannon et al., 2003) using the Metscape (Karnovsky et al., 2012) plugin. Compounds displayed represent members of metabolic pathways identified as significantly enriched by Mummichog analysis.

### Correlation Analysis

The correlation analysis of metabolite abundance and viral titer was perfomed in R version 4.0.2 (R Core Team, 2003) using the following packages: corrr correlation analysis (Kuhn et al., 2020), dplyr (Wickham et al., 2020) tidyr (Wickham and Henry, 2020), ggplot2 (Wickham, 2016), and viridis (Garnier, 2018). Compounds included in the correlation analysis were those with significant shifts in abundance relative to ZIKV infection status (FDR < 0.05).

### Inosine Assay

To confirm changes in inosine concentration upon viral infection a secondary biochemical assay was performed to verify the metabolomic predictions. The analysis was perfomed using a commercial inosine assay kit (Sigma-Aldrich, MO, United States). The assay utilizes a coupled enzyme reaction in which inosine is converted to hypoxanthine which reacts with the substrate mix and probe resulting in a fluorometric product (⁁_ex_ = 535 nm/⁁_em_ = 587 nm). The resulting absorbance is proportional to the inosine abundance. Mosquitoes were reared and offered a blood meal as described above and 7 days post infectious blood meal, the mosquito’s legs were dissected and placed in a labeled vial and the whole body transferred to a separate vial. The legs of the mosquitoes were screened for ZIKV infection as described above to ascertain infection.

Six singleton female mosquitoes exposed to ZIKV infectious and non-infectious blood meals were homogenized in 0.7 ml of ice-cold 0.4 M perchloric acid and centrifuged at 13,000 × *g* for 10 min in order to remove the insoluble material. The supernatant was transferred to a new tube and then neutralized by adding ice cold 70 μl of 4M potassium carbonate solution. The solution was incubated on ice for 7–10 min and then centrifuged at 13,000 × g for 10 min.

Each sample was then diluted 1:10 and 1 μl of both diluted and undiluted sample were added to the wells of black plates with clear bottom. The samples were brought to a final volume of 50 μl with inosine assay buffer and 50 μl of the reaction mix was added to each well including the inosine standards and the sample blank. The fluorescence intensity was measured at ⁁_ex_ = 535 nm/⁁_em_ = 587 nm. The amount of inosine present in the samples was determined from the standard curve. Differences in the concentration of inosine across the infection status was compared by *T*-test.

## Results

### Global Metabolic Profile Analysis

A total of 209 *Ae. albopictus* individuals were exposed to 8.3 log_10_ PFU/ml ZIKV HND strain infectious blood meal, the overall infection rate of exposed individuals was 26%, with an average of Log_10_ 7.64 copy number standard ZIKV and a standard deviation of ±0.89 (Supplementary File S1).

Samples were subjected to untargeted metabolic analyses screening for primary metabolites (carbohydrates, proteins, amino acids, vitamins, acetone, and ethanol), lipids (fats, phospholipids, vitamins, and steroids) and biogenic amines (monoamine neurotransmitters and polyamines). Only features (molecular identities defined by a unique mass to charge ratio, m/z and retention time) detected in all the 20 samples (representing 10 infected and 10 uninfected) were considered ‘present’ in the samples. Comparison of ZIKV infected versus uninfected mosquitoes revealed clusters of metabolites showing significant differences in abundance between infected and uninfected mosquitoes.

A total of 473 primary metabolites were detected, however, after filtering and normalization, 425 were analyzed. Of these, 29% were identified at the structural level, while the rest remain unidentified. Of the identified primary metabolites, 41 show differential abundances between treatment groups using a log fold change ≥ 2 and *T*-test/FDR *P*-values ≤ 0.05 being considered differential.

After normalization, 1809 lipids and lipid related compounds were detected and 409 were identified to the structural level. Of these, 167 showed differential abundances based on the same statistical parameters as used for the primary metabolites. Of the compounds detected in the biogenic amines panel, 896 were detected with 28% of those identified to structure. Of those, 26 met the threshold of differential abundance. Analysis of the detected compounds from the three panels by partial least squares – discriminant analysis (PLS-DA) revealed a clear separation between uninfected and infected samples and highlighted an array of compounds correlating with infection state. ZIKV-infection distinguished the primary metabolite, identified and unidentified lipids and biogenic amines when plotted together.

### Metabolites Associated With RNA Editing and Glucose Levels Reduction Is Observed in ZIKV-Infected *Ae. albopictus*

**FIGURE 1:**
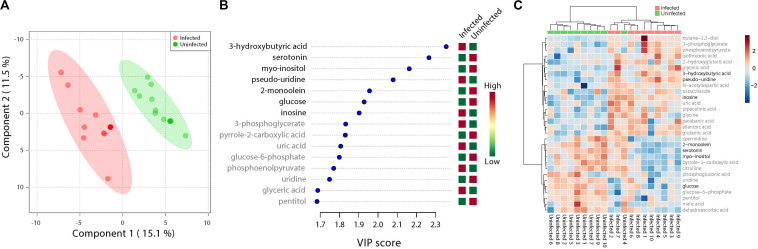
Fluctuations in levels of primary metabolites following ZIKV infection of mosquito whole bodies. ZIKV infection is associated with alterations in primary metabolites abundance. **(A)** Multivariate comparative analysis performed using partial least squares discriminant analysis (PLS-DA) component biplot revealed a distinct clustering of primary metabolites according to the infection status of the individual source. **(B)** Variable Importance in Projection (VIP) score, a measure of a metabolites’ importance in the model is reported for each of the top 15 metabolites. This measure is calculated by removing the relationship of a metabolite and measuring increase in error. The metabolite with highest VIP score is considered to have the highest association with the infectious state. The *X* axis indicates the VIP scores corresponding to each variable on the *Y* axis. Red boxes indicate metabolites increased abundance in infected replicates while green indicates decreased abundance. The metabolites represented in black fonts represent that which were statistically significant by *t*-test (FDR < 0.05). **(C)** Heatmap plot was generated on the MetaboAnalyst program using hierarchical, agglomerative cluster analysis. The distance measure was Euclidean (Motzkin, 1949) and the clustering algorithm utilized was Ward (Ward and Hook, 1963). The *t*-tests were performed with a cut off of an FDR adjusted *p*-value of 0.05. The *X* axis represents the individuals with the corresponding infection status while the *Y* axis represents the compounds identified with the statistically significant (FDR < 0.05) compounds represented in black. The heat map shows the level of intensity of abundance of each compound across each individual. Red coloration represents increased abundance while blue coloration represents decreased abundance in relation to infection state.

**FIGURE 2:**
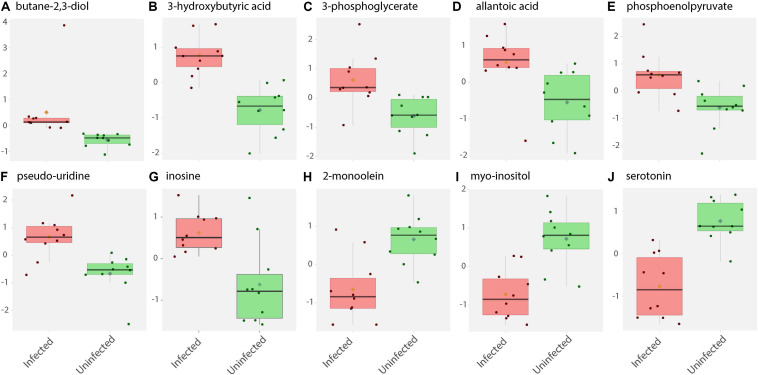
Box and whisker plots of biochemical abundance of metabolites in ZIKV-infected *Ae. albopictus* whole bodies. Untargeted metabolic analysis was utilized to screen for primary metabolites in *Ae. albopictus* females infected with ZIKV (ZIKV- positive legs). The metabolites were profiled using Gas Chromatography time of flight (GC TOF) protocol. The data was analyzed using the MetaboAnalyst (v4.0) package. Each plot **(A–J)** represents an individual compound as labeled. Compounds were selected based on fold change analysis with threshold log fold change values ≥ 2 and an FDR adjusted *p*-value of < 0.05. The individuals exposed to ZIKV infected blood meal (8.3 log_10_ PFU/ml ZIKV HND strain) are represented by a pink box and whisker plot while individuals exposed to non-infectious blood meal are represented by a green box and whisker plot.

**FIGURE 3:**
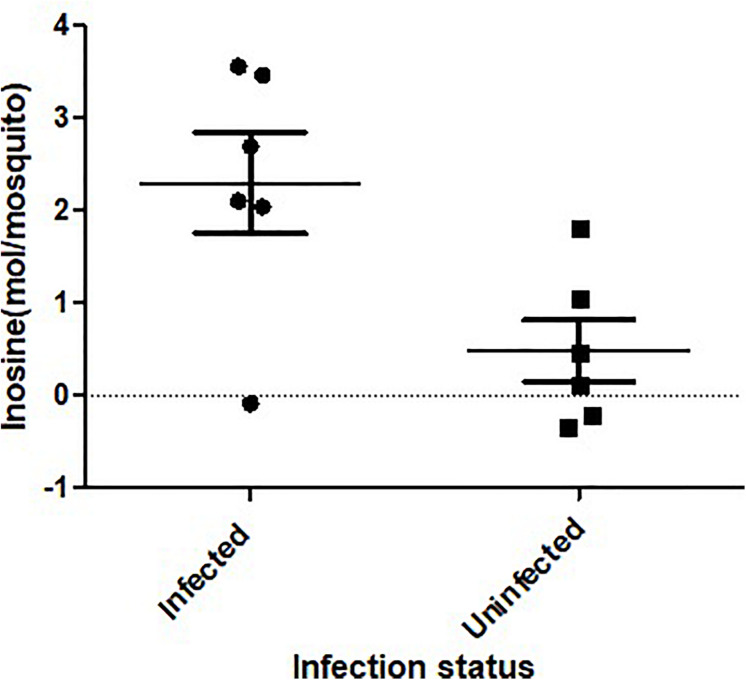
Inosine quantification in ZIKV-infected *Ae. albopictus*. A fluorometric assay was utilized to compare inosine levels in ZIKV infected (ZIKV- positive legs) and non-infected *Ae. albopictus* individuals. The individuals were offered 8.3 log_10_ PFU/ml ZIKV HND strain blood meal or uninfected blood meal. At 7 dpi, the legs were dissected and screened for infection and the whole body of individual mosquito was used to determine the inosine levels. The background was corrected by subtracting the blank fluorescent reading value from all readings and the standard curve generated from the corrected values of the inosine standards. The sample blank value was subtracted from the sample readings and the amount of inosine present was determined from the standard curve using the corrected measurements. The *X* axis represents the infection status of the mosquitoes while the *Y* axis represents the concentration (moles) per mosquito.

ZIKV infection resulted in an enrichment of levels of metabolites associated with RNA editing as well as a perturbation of glucose metabolism levels. RNA editing catalyzed by enzymes of the adenosine deaminase acting on RNA (ADAR) family converts adenosine to inosine in a double-stranded RNA (Zinshteyn and Nishikura, 2009). Additionally, pseudo-uridine, an isomer of the nucleoside uridine, occurs in rRNA, tRNA and small nuclear and nucleolar RNA but not in mRNA or viral genomic RNAs (Carlile et al., 2019). We measured a significant increase in pseudo-uridine abundance among the pools of *Ae. albopictus* exposed to infectious blood meals (T – stat 4.165; *P* = 0.0005; FDR = 0.019781) (Figures 1B,C, 2F). Inosine, a purine nucleotide is found at the wobble position of tRNA, playing a role in the proper translation of mRNA at the ribosome. Apart from its role in translation, it plays a role in the immune system exhibiting both inflammatory and anti-inflammatory effects. We measured the impact of ZIKV infection of *Ae. albopictus* on the levels of inosine. Further, increased abundance of inosine was also detected in the infected groups (T – stat 3.5434; P = 0.002; FDR = 0.005) (Figures 1B,C, 2G, 3). We carried out a separate inosine assay experiment to determine the impact of ZIKV infection on inosine levels of *Ae. albopictus*. We observed an enrichment of inosine among ZIKV in *Ae. albopictus* (*t*-test *P* = 0.0178; *t* = 2.832 df = 10) (Figure 3).

Specifically, levels of phosphoenolpyruvate, [responsible for generation of adenosine triphosphate (ATP), a major source of chemical energy within cells], were increased among infected samples relative to the uninfected (T – stat 3.5845; *P* = 0.002; FDR = 0.04) (Figure 2E). In addition, a significant increase of 3- phosphoglycerate metabolites among the infected individuals was noted (T – stat 3.9136; *P* = 0.001; FDR = 0.02) (Figures 1B,C, 2C), allantoic acid, involved in uric acid degradation was upregulated among ZIKV-infected individuals (Figure 2D), 3- phosphoglycerate is an important component of the glycolysis pathway. Levels of the ketone body 3- hydroxybutyrate, were also elevated (T – stat 5.5305; *P* = 2.99E-05; FDR = 0.0040654) (Figures 1B,C, 2B). This metabolite is generated during the metabolism of fatty acids (e.g., butyrate) and is usually synthesized to provide backup energy in the case of reduced glucose levels (Newman and Verdin, 2017). The infected groups appear to demonstrate increased energy expenditure as evidenced by reduced levels of glucose (T – stat -3.6259; *P* = 0.0019; FDR = 0.04) (Figures 1B,C) and glucose -6- phosphate (Figures 1B,C), a major intermediate in the mobilization of glucose as an energy source. This is a potential indicator that infected mosquitoes are undergoing increased rates of glycolysis in association with viral replication. Both myo-inositol and butane- 2,3- diol, products of glucose metabolism demonstrated significant change in abundance due to ZIKV infection (Figure 2I). Butane- 2,3 – diol, a volatile compound associated with certain strains of root-associated bacteria (Kong et al., 2018) and capable of being produced from glucose (Chu et al., 2015) was highly abundant (*P* = 0.013; Log_2_^5^ fold change) in the infected samples (Figure 2A).

Serotonin as a neurotransmitter controls multiple processes related to feeding and nutrition in mosquitoes (Moffett and Moffett, 2005). Results of this study analysis revealed that infected mosquitoes show a significant decrease in serotonin levels (T – stat −5.0104; *P* = 9.08E-05; FDR = 0.0062) indicating that the infection may be having an effect on the serotonergic system (Figures 1B,C, 2J). In addition, 2-monoolein, a monoglyceride linked to cell membrane fluidity, was downregulated in the infected tissues (T – stat −3.7195; *P* = 0.002; FDR = 0.04) (Figures 1B,C, 2H) (Singh et al., 2019). Apart from the above-mentioned samples, there were an array of metabolites whose abundance were differentiated but at an insignificant level. These includes metabolites such as uric acid, glyceric acid, pentitol, malic acid, and many others.

### Enrichment of Arachidonic Acid Observed in ZIKV-Infected *Ae. albopictus*

**FIGURE 4:**
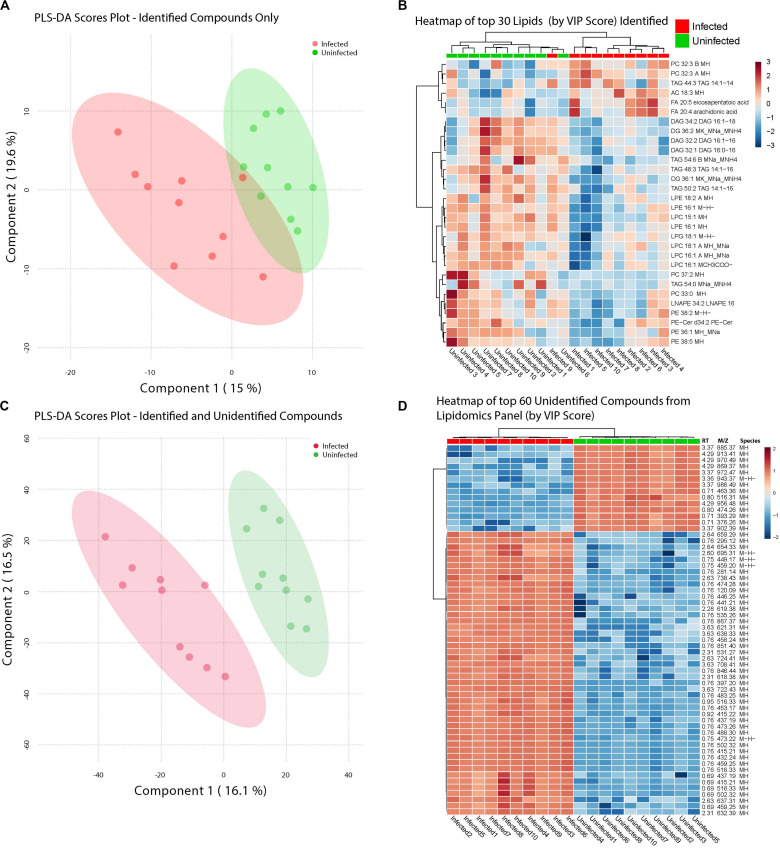
ZIKV-infection results in alteration of the Lipid homeostasis in infected *Ae. albopictus*. The lipid levels alteration due to ZIKV infection were analyzed by multivariate comparative analysis using partial least squares discriminant analysis (PLS-DA). **(A)** Biplot of components 1 and 2 of analysis including LC-MS identified compounds only. **(B)** Heatmap of top 30 identified lipids as determined by VIP score. **(C)** Biplot of components 1 and 2 of analysis including all detected compounds. **(D)** Top 60 compounds (identified and unidentified) as determined by VIP score from LC-MS based lipidomics panel. Unidentified compounds are labeled by m/z (mass/charge ratio) and RT (retention time) and species. Heatmap aesthetics are equivalent to those in Figure 1.

Infection with ZIKV differentiated the lipids present in samples of *Ae. albopictus*. This was observed both when Multivariate comparative analysis was done using identified compounds as well as identified and non-identified compounds (Figures 4A,B). A large number of the lipids whose levels were altered due to ZIKV infection have not been previously identified, however, they demonstrated a clear profile of abundance in association with the infection status of the individuals (Figures 4B,D).

**FIGURE 5:**
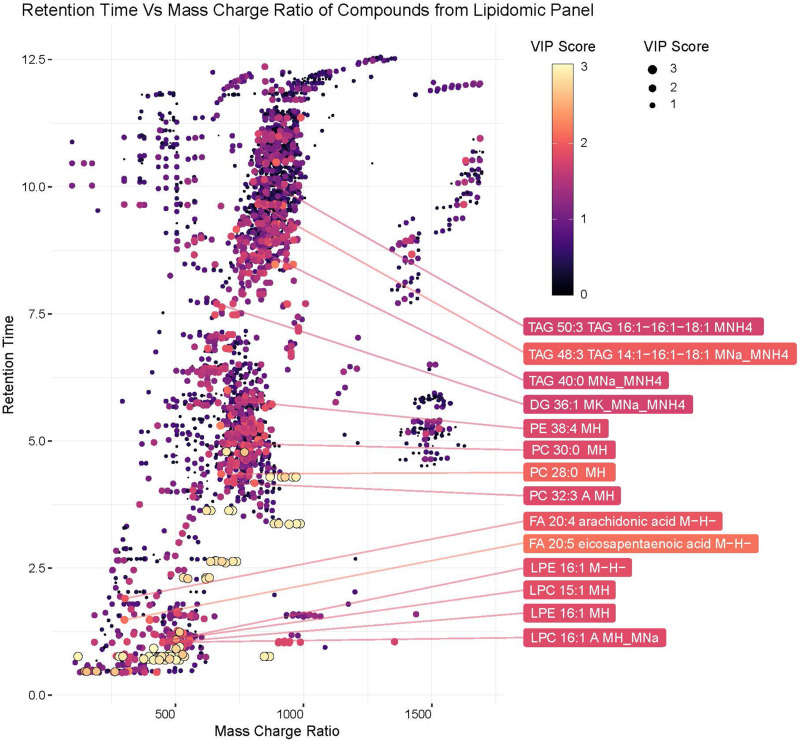
Plot of hydrophobic compounds identified by LC/MS based on mass/charge ratio and retention times. To visualize compounds with common physical features associated with significant compounds, mass/charge ratio was compared against the Gas Chromatography Column retention time. The Identified lipids within the top 200 VIP scores were annotated. The VIP scores for each compound are represented by the color and size of each point.

Lipids play critical roles in multiple stages in the virus replication cycle and the unique induction of a lipid profile upon viral infection is required for optimal virus replication (Diamond et al., 2010; Heaton and Randall, 2010; Peña and Harris, 2012; Perera et al., 2012; Martin-Acebes et al., 2014; Xu and Nagy, 2015; Melo et al., 2016; Chotiwan et al., 2018). Infected mosquitoes showed multiple perturbations in their lipid constitution. In particular, arachidonic acid, a precursor of eicosapentaenoic acid (Eijkelkamp et al., 2018) (Figures 4, 5) was differentially expressed due to ZIKV infection. When the unidentified compounds were included in the analysis, some show a highly significant correlation with ZIKV infection status. Both the identified (arachidonic acid and eicosapentaenoic acid) were plotted based on their mass/charge ratio against the Gas Chromatography Column Retention Time. The lipids within the top 200 VIP scores were annotated and they appeared to cluster in a similar space as the short chain lysophospholipids demonstrating a potential for shared properties in terms of M/Z and retention time (Figure 5).

### ZIKV Infection Results in the Differential Abundance of Metabolites Associated With Immunomodulatory Roles

**FIGURE 6:**
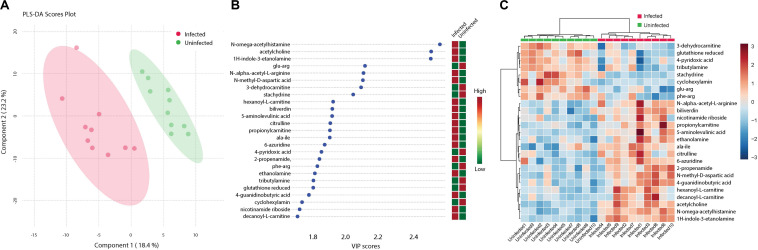
Cluster analysis of biogenic amine levels differentiation due to ZIKV infection. Biogenic amines are low molecular weight organic compounds formed by decarboxylation of amino acids. **(A)** Clustering of biogenic amines using partial least squares’ discriminant analysis (PLS-DA) score plot after ZIKV infection of *Ae. albopictus* metabolites reveal a distinct intensity profile that can distinguish the ZIKV- infected from the uninfected *Ae. albopictus* individuals. **(B)** The color spectrum indicates the level of each compound Red indicates high intensity while green, low intensity. The VIP score indicates the level of importance of the biogenic amine to variance between treatment groups. **(C)** A heatmap was used to cluster the biogenic amines across the different infection status. Heatmap aesthetics are equivalent to those in Figures **1C**, **4C**.

Analysis of metabolites identified among ZIKV-infected and uninfected *Ae. albopictus* individuals resulted in defined clusters of biogenic amines associated with either infected or uninfected *Ae. albopictus*. Biogenic amines are low molecular weight organic compounds formed by decarboxylation of amino acids. They are known to modulate energy metabolism in insects and regulate the secretion of other hormones (Hirashima et al., 2000). They also function as neurotransmitters and mediate cross talk between the nervous and immune systems (Sternberg, 1997). Acetylcholine, known for its role in neurotransmission (Picciotto et al., 2012) and immunity (Rajendran et al., 2015) is increased in abundance among the infected samples (T – stat 7.9272; *P* = 2.79E -07; FDR = 2.44E-05) (Figure 6B). In addition, both 6- azuridine (T – stat 3.7079; *P* = 0.002; FDR = 0.03) and biliverdin (T – stat 3.8715; *P* = 0.001; FDR = 0.02) were upregulated among the infected individuals (Figures 6B,C). Stachydrine (T – stat -4.3757; *P* = 0.0004; FDR = 0.01) as well as 3-dehydrocarnitine (T – stat −4.5924; *P* = 0.0002; FDR = 0.008) (an intermediate in the production of carnitine) were significantly depleted in infected individuals (Figures 6B,C). Increased levels of *N*-methyl-D-aspartic acid were observed among the infected individuals (T – stat 4.6483; *P* = 0.0002; FDR = 0.008) (Figures 6B,C). *N*-alpha-acetyl-L-arginine levels were enriched among infected individuals (T – stat 3.8675; *P* = 0.001; FDR = 0.02). L-arginine may inhibit glycation and advanced glycosylated end product formation (Servetnick et al., 1996). Increased 5-aminolevulinic acid levels were measured among ZIKV-infected individuals (T – stat 4.6483; *P* = 0.0002; FDR = 0.008). It is a product of glycine and succinyl CoA and its synthesis is regulated by the amount of heme in the cell (Peng et al., 1997). ZIKV infection was associated with enrichment of Citrulline (T – stat 3.828; *P* = 0.001; FDR = 0.02). Citrulline is important for protein biosynthesis and normally, ornithine is converted to citrulline before conversion to arginine (Windmueller and Spaeth, 1981). In addition, propionyl-carnitine, a biogenic amine known to possess superoxide scavenging and antioxidant properties (Vanella et al., 2000) was enriched among the infected individuals (T – stat 3.8197; *P* = 0.001; FDR = 0.02) (Figures 6B,C).

### Pathway and Network Analysis

**FIGURE 7:**
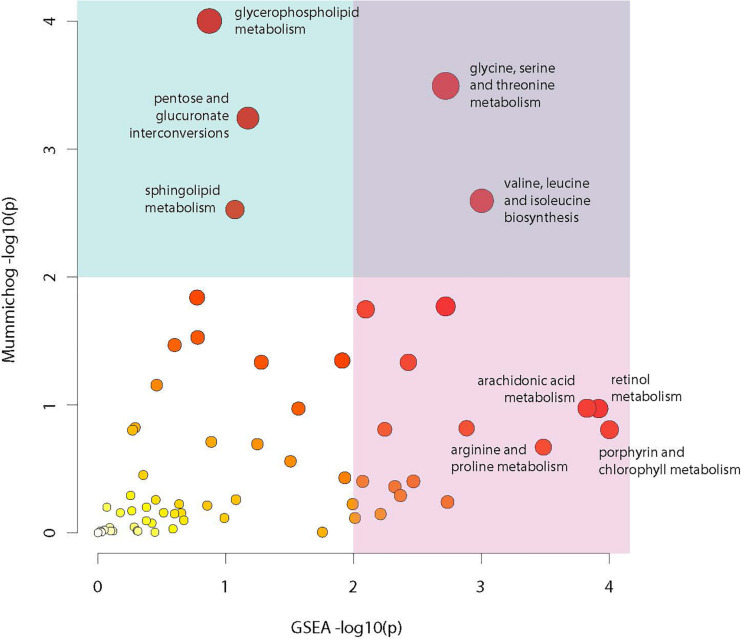
Metabolic pathway enrichment. Visualization of enriched metabolic pathways as identified using the Mummichog pathway analysis package. Analysis utilized raw mz and rt values for LC-MS profiles from the lipidomic and biogenic amine analysis panels in combination with *p*-values from statistical comparisons between treatment groups to identify pathways with perturbations in compound abundances in infected mosquitoes. The *X* axis represents −log10 *p*-value derived from the GSEA (Gene Set Enrichment Analysis) algorithm and the *Y* axis represents the −log10 *p*-value derived from the Mummichog enrichment analysis algorithm. Size and color of dots represents significance.

Analysis of compounds present in the lipidomic and biogenic amine datasets for enrichment of specific biochemical pathways revealed statistically significant abundance shifts across multiple pathways. Identified pathways include: glycine, serine and threonine metabolism; valine, leucine and isoleucine biosynthesis; glycerophospholipid metabolism; arachidonic acid metabolism; retinol metabolism; pentose and glucuronate interconversion; sphingolipid metabolism; arginine and proline metabolism as well as porphyrin and chlorophyll metabolism (Figure 7). Metabolites from these pathways along with their relative abundances and statistical values were visualized to identify relationships between these pathways and to highlight large changes in metabolite abundance.

**FIGURE 8:**
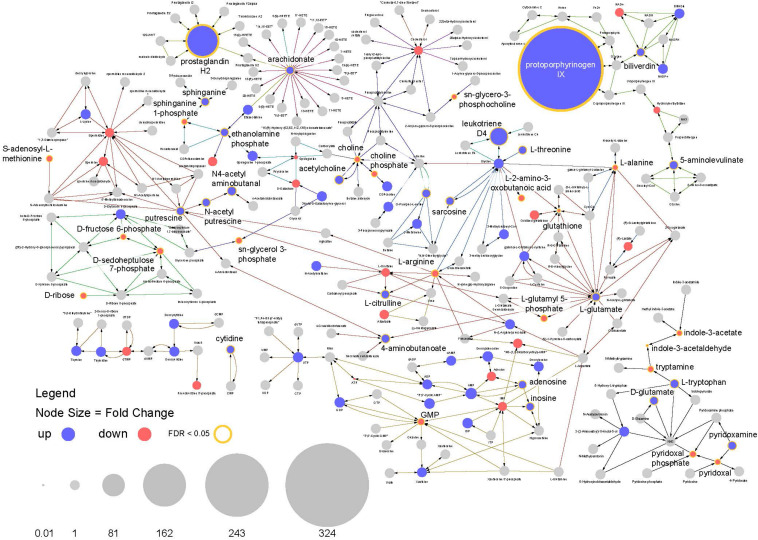
Pathway analysis. Visualization of relative compound abundances and statistical significance from pathways identified by pathway enrichment analysis. Points represent individual compounds. Blue points are more abundant in ZIKV infected mosquitoes and red points are less abundant. Point sizes represent the fold change relative to uninfected mosquitoes. Yellow borders around points and increased label font size represent significant changes in compound abundance (FDR < 0.05). Arrows represent enzyme mediated conversions of one compound to another.

This analysis highlights multiple features of note in infected mosquitoes. The most prominent feature is a >300-fold increase in the compound protoporphyrinogen IX (a known antiviral that blocks viral cell invasion) (Paiva-silva et al., 2006) (Figure 8). This compound is synthesized from biliverdin which is a byproduct of heme metabolism in blood feeding insects and also shows a significant increase in abundance in infected mosquitoes.

Another observation is a 100-fold increase in abundance of prostaglandin H2, which is the precursor to all prostaglandin biosynthesis. In connection with this, compounds required for prostaglandin synthesis (including arachidonate, ethanolamine phosphate and sphinganine) were also significantly enriched among infected individuals (Figure 8). These findings suggest the arachidonic acid pathway is playing an important role in the infection response and facilitating increased prostaglandin synthesis.

In addition, a large increase in the abundance of leukotriene D4 was observed in infected mosquitoes along with perturbations in other compounds associated with its biosynthesis. As with prostaglandins, leukotrienes are also associated with inflammatory responses to infection and allergens in vertebrates (Figures 7, 8).

**FIGURE 9:**
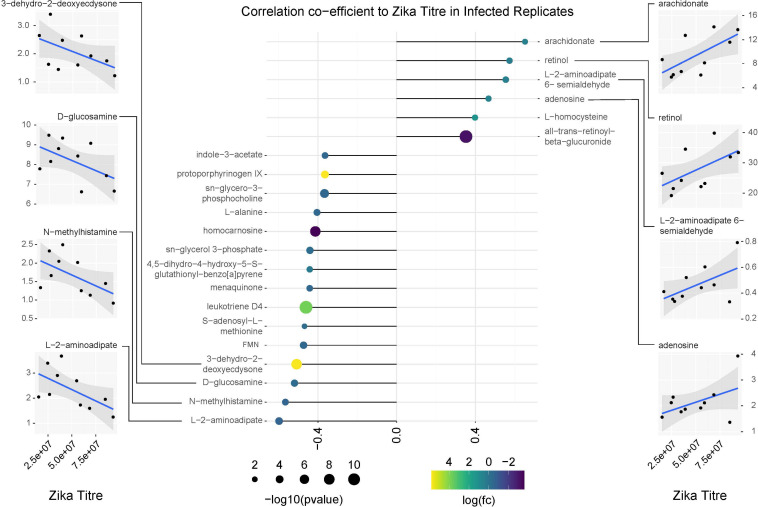
Correlation analysis of compounds associated with ZIKV infection relative to replicate infection titers as measured by qPCR. Lollipop chart visualizing correlation coefficients of compounds with significant changes in abundance in ZIKV positive mosquitoes. Compound abundance was compared against the average ZIKV titer from each biological replicate of infected samples. Only compounds with a correlation coefficient outside of –0.35 or 0.35 were plotted. Point size represents the significance of the change in compound abundance between infected and uninfected samples. Point coloration represents log of the fold change observed in infected samples. Individual plots for compounds with the highest positive and negative correlations relative to ZIKV titer are found on the left and right sides of the figure. Blue trendlines represent general linearized models of the correlation. Shaded areas represent 95% confidence intervals.

Arachidonic acid metabolism pathway was found to be significantly enriched among infected individuals as well as three prostaglandins (prostaglandin E2, prostaglandin H2 and (5Z,13E)- (15S)-9alpha,15-dihydroxy-11-oxoprost-5,13-dienoate, that showed very high abundance in the infected flies (Figures 7, 9). The prostaglandins are likely derived from the arachidonic acid as that feeds into the prostaglandin synthetic pathway.

## Discussion

This study observed significant differences in metabolites between uninfected and ZIKV infected mosquitoes. These include differential abundances of pseudo-uridine and inosine metabolites which are associated with post transcriptional RNA modification activities. This was confirmed in a separate experimental assay showing significant enrichment in the levels of inosine among infected *Ae. albopictus*. RNA-editing enzymes, Adenosine deaminases that act on RNA (ADARs) deaminates double stranded RNA (dsRNA) converting adenosine (A) to inosine (I) in pre-mRNA hairpins (Bass and Weintraub, 1988). The conversion of A to I is read by the translation machinery as guanosine hence altering the protein sequence that’s encoded by edited mRNA (Polson and Bass, 1994). *In silico* analysis using complete coding sequences of the ZIKV polyprotein have demonstrated that host-mediated RNA editing of adenosines (ADAR) play a role in molecular evolution of ZIKV (Piontkivska et al., 2017). Further, most of the ADAR-edited transcripts are expressed in the central nervous system of *Drosophila* and hypothesized to result in more than one protein produced from a single gene (Palladino et al., 2000). In fact, a nuclease has been shown to cleave edited dsRNA in *Drosophila* demonstrating that ADARs may be involved in tagging viral RNA for degradation (Carpenter et al., 2009). Further, ADAR has been shown to result in hyper-editing in the sigma virus, a negative sense RNA virus that is a pathogen of *D. melanogaster* (Carpenter et al., 2009). ADAR is considered a part of the antiviral response, indeed the loss of *Adar* RNA editing induces the expression of immune-related genes in *Drosophila* (Carpenter et al., 2009). In order to establish causality between ZIKV infection and post transcriptional modification due to ADAR expression, future mechanistic studies are necessary.

In this study, ZIKV infection of *Ae. albopictus* resulted in hypoglycemia and an enrichment of phosphoenolpyruvate and ketone body 3- hydroxybutyrate which is an energy source produced when glucose is not readily available (Laffel, 1999). Energy depletion would be a consequence of a direct competition of resources between the mosquito and ZIKV (Maier et al., 1987) or the infected mosquito would require extra nutrients to restore damaged tissues or even to fuel the cost of mounting immune response (Ferdig et al., 1993; Ahmed et al., 2002). The results of our study corroborates the findings of Shrinet et al. (2018) which demonstrated that glycolysis/gluconeogenesis played an important role in both mono and co-infection of *Ae. aegypti* mosquitoes with CHIKV and DENV. In addition, using a proteomics differential approach with two-Dimensional differential in-Gel Electrophoresis (2D-DIGE), Tchankouo-Nguetcheu et al. (2010), studied the protein modulations in the midgut of *Ae. aegypti* after seven day oral infection with DENV-2 and CHIKV viruses. The study demonstrated that enzymes participating in the glycolytic pathway were upregulated by CHIKV or DENV-2 suggesting extensive glucose utilization during midgut infection.

Serotonin levels were decreased among the infected individuals. Serotonin is a neurotransmitter and its immunoreactive neurons which innervate the chemosensory systems of mosquitoes suggest that serotonin is a neuromodulator in the chemosensory processes of disease vector mosquitoes (Siju et al., 2008). In fact, serotonin depletion in *Aedes triseriatus* resulted in low blood feeding levels but did not affect host seeking behavior (Novak and Rowley, 1994) and in *Ae. aegypti*, low serotonin levels resulted in prolonged probing as well as a lower blood feeding success rate (Novak et al., 1995). A decrease in serotonin levels may be indicative of the modulation of insect behavior and fitness effect of ZIKV infection.

Lipids in both the viral envelope and host cell membranes play important roles throughout the attachment process during viral infection (Lorizate and Kräusslich, 2011). In this study, a significant number of lipids that were significantly enriched due to ZIKV infection have not been previously determined. Our findings on lipid perturbations due to ZIKV infections corroborates the findings of Chotiwan et al. (2018), study which demonstrated that upon DENV infection of *Ae. aegypti*, a majority of lipids that showed greater than 2 -fold changes in intensity had higher abundances upon DENV infection.

Fatty acids play a very important role in virus protein synthesis as well as replication (Cordero et al., 2014). In this study, both eicosapentaenoic and arachidonic acid were upregulated among the infected individuals. The enzymatic oxidation of arachidonic acid results in the production of prostaglandin (Black et al., 1980). Our study demonstrated that infection of *Ae. albopictus* with ZIKV resulted in enriched levels of prostaglandins including prostaglandins H2. Prostaglandin A1 reduced Mayaro virus replication in *Ae. albopictus* mosquito cells in culture by inhibiting virus-specific protein synthesis while in mock infected cells, the PGA1 stimulated the synthesis of several proteins (Barbosa and Rebello, 1995). Future mechanistic studies aimed at understanding the impact of prostaglandin on the immune priming of mosquitoes could result in novel transmission barrier mechanisms.

In our study, when we set the P cut-off value at *P* ≤ 0.1, we measured a decrease of phosphatidylethanolamine (PE), important for the biophysical properties of membranes such as fluidity and curvature (Xu and Nagy, 2015). Indeed, lipid metabolites vital to virus assembly such as triacylglycerols (TAG) (Liefhebber et al., 2014) consisted the highest abundance relative to other compounds within the lipidome. Phosphatidylethanolamine (PE) and phosphatidylcholine (PC) were the second and third most abundant lipids respectively, phosphatidylcholine (PC) was increased in abundance among the infected individuals. The significance was lost when we decreased the *P*-value threshold to ≤0.05. The results of our studies differ from the results obtained from a study on lipid composition after DENV infection whereby PC, PE and phosphatidylserine levels were significantly enriched upon DENV infection of insect cell lines (Perera et al., 2012). In addition, glycerophospholipids, sphingolipids and fatty acyls were enriched in *Ae. aegypti* mosquitoes infected with DENV (Chotiwan et al., 2018). Further, our study findings differ from that of a study that utilized a whole cell lipidomics approach seeking to identify the cellular lipids important for WNV_KUN_ replication. This study observed an elevated level of phospholipase A2 as well as lyso phosphatidylcholine (Liebscher et al., 2018). We cannot rule out differences in pathogen vector host interactions as well as disease models utilized as causing the different results between these studies.

Further, biliverdin levels was upregulated among ZIKV-infected *Ae. albopictus*. Hemoglobin, the main blood protein is digested by mosquitoes to produce large amounts of heme which is further digested by heme oxygenase to produce biliverdin IX alpha, CO and free iron (Bottino-Rojas et al., 2019). Heme increases the microbial load in the mosquito midgut which has an antagonistic effect on DENV infection (Xi et al., 2008; Ramirez et al., 2012). In addition, heme also directly suppresses mosquito immune activity leading to a higher susceptibility of mosquitoes to DENV infection (Sim et al., 2013). Our study documents a > 300-fold increase in the compound protoporphyrinogen IX. This compound is synthesized from biliverdin. The antiviral properties of natural and synthetic porphyrins have been demonstrated (Staudinger et al., 1996; Vzorov et al., 2002; Chen-collins et al., 2003; Benati et al., 2009; Assuncao-Miranda et al., 2016). It has been hypothesized that the mechanism involved in viral inactivation by porphyrins to be dependent on its interaction with viral particles. In fact, DENV replication was inhibited only when porphyrins were in direct contact with newly formed particles or in infection with very low multiplicity of infection (MOI). Further porphyrins were shown to be capable of inhibiting the early stages of DENV infection such as adsorption and penetration in susceptible cells (Assuncao-Miranda et al., 2016).

Though its primary role is to protect organisms from infection and injury, an overactive immune response can result in excessive inflammation and damaged organs. We measured an increase in levels of acetylcholine in ZIKV-infected individuals, suggesting it may have proviral function during ZIKV infection. Understanding how acetylcholine may modulate the immune pathways will be important for understanding viral tropism in mosquitoes.

In addition, in this study, histidine metabolism pathway was significantly perturbed by ZIKV infection with an enrichment of L-histidine and imidazole-4-acetaldehyde. Imidazoles are heterocycles with five-member ring structure. Imidazoles have gained importance due to their exceptional pharmacological activities (Kumar et al., 2017). In a search for new compounds with potential for clinical use as antiviral agents, high throughput screening of a compound library resulted in the identification of imidazole-4,5-dicarboxamide derivative and demonstrated it to have a high DENV inhibitory activity (Saudi et al., 2014). Future mechanistic studies to clarify the role of imidazole-4-acetaldehyde in ZIKV infection is important.

In summary, elevation of pseudo-uridine and inosine in ZIKV-infected *Ae. albopictus* may be indicative of a post-transcriptional modification of RNAs as a result of ZIKV infection. However, mechanistic studies to establish causality is important. Future studies targeting select differentiated metabolites for targeted metabolic studies is very important in order to validate the observations made by this study. Understanding the interactions between proviral pseudo-uridine, inosine and ZIKV replication will impact our understanding of the process and pathology of ZIKV. Further, the results of our study suggest that infected mosquitoes may be impacted by negative fitness effects that increase transmission efficiency. The reduced levels of serotonin may affect salivary gland function resulting in decreased feeding efficiency and increased vector/host contact. The metabolites responsive to ZIKV infection identified in this study may represent an immune response by the mosquito and in future, upon validation may act as points to prevent ZIKV transmission. Overall, these findings provide an array of insights into the biochemical interactions occurring during ZIKV infection in *Ae. albopictus* and provide potential targets that can inform the development of novel intervention strategies.

## Data Availability Statement

All datasets generated for this study are included in the article/Supplementary Material.

## Author Contributions

MO was involved in the study design, conducted the experiments, and drafted the manuscript. GA was involved in the study design, analyzed the results, and participated in the writing of the manuscript. SB, JS, and EB were involved in the mosquito rearing and assisted in mosquito manipulation exercises. EK was involved in data analysis and visualization. LK was generated and propagated the ZIKV infectious clone utilized in this study. AC and LK were involved in the study design and participated in the writing of the manuscript. All the authors contributed to the article and approved the submitted version.

## Conflict of Interest

The authors declare that the research was conducted in the absence of any commercial or financial relationships that could be construed as a potential conflict of interest.

## Supplementary Material

Supplementary File 1 An excel file showing the pooling of the *Ae. albopictus* samples used in the metabolomics study and demonstrating the ZIKV copy number standards in each individual associated with each of the pool.

Supplementary File 2 A table listing the internal standards included in the lipidomic and biogenic amine panels.

Supplementary File 3 Raw GC-MS peak values with annotations from primary metabolites panel.

Supplementary File 4 Raw LC-MS peak values with annotations from lipidomics panel.

Supplementary File 5 Raw LC-MS peak values with annotations from biogenic amines panel.

Supplementary File 6 Primary metabolite GC-MS peak data formatted for Metaboanalyst submission.

Supplementary File 7 Lipidomics LC-MS peak data formatted for Metaboanalyst submission.

Supplementary File 8 Biogenic amine LC-MS peak data formatted for Metaboanalyst submission.

Supplementary File 9 Combined Lipidomics and Biogenic amine LC-MS data formatted for submission to Peaks-to-Pathways (Mummichog) package on Metaboanalyst.

Supplementary File 10 Pathway enrichment data output from Peaks-to-Pathways.

Supplementary File 11 Compound KEGG identifications output from Peaks-to-Pathways.

Supplementary File 12 Excel workbook containing the normalized combined lipidomic and biogenic amine LC-MS datasets annotated with KEGG ids for correlation analysis and pathway analysis in Cytoscape.
