# Corrigendum: Zika Virus Infection Results in Biochemical Changes Associated With RNA Editing, Inflammatory and Antiviral Responses in *Aedes albopictus*

**DOI:** 10.3389/fmicb.2020.642886

**Published:** 2021-01-15

**Authors:** Maria G. Onyango, Geoffrey M. Attardo, Erin Taylor Kelly, Sean M. Bialosuknia, Jessica Stout, Elyse Banker, Lili Kuo, Alexander T. Ciota, Laura D. Kramer

**Affiliations:** ^1^Wadsworth Center, New York State Department of Health, Slingerlands, NY, United States; ^2^Department of Entomology and Nematology, University of California, Davis, Davis, CA, United States; ^3^School of Public Health, State University of New York, Albany, NY, United States

**Keywords:** Zika virus, *Aedes albopictus*, primary metabolites, lipids, biogenic amines, metabolomic phenotyping

In the published article, there was an error regarding the affiliations for Sean M. Bialosuknia. Instead having affiliations 1,2,3, they should have affiliations 1,3.

In the original article, we neglected to include funding from Pacific Southwest Regional Center of Excellence for Vector-Borne Diseases funded by the U.S. Centers for Disease Control and Prevention (Cooperative Agreement 1U01CK000516) to Erin Taylor Kelly and Geoffrey M. Attardo.

## Funding

This publication was supported by the Cooperative Agreement Number U01CK000509 funded by the Center for Disease Control and Prevention. Its content is solely the responsibility of the authors and do not necessarily represent the official views of the Center for Disease Control and Prevention or the Department of Health and Human Services. The study received funding from Pacific Southwest Regional Center of Excellence for Vector-Borne Diseases funded by the U.S. Centers for Disease Control and Prevention (Cooperative Agreement 1U01CK000516), to EK and GA. We would like to extend our gratitude to Illia Rochlin of Suffolk County Health Department for providing us with Ae. albopictus mosquitoes and the Arbovirus laboratory insectary crew for mosquito maintenance. Data for these analyses were acquired at the University of California, Davis West Coast Metabolomics Center, U2C ES030158. We would like to express our gratitude to Dr. Alice Trimmer for her suggestions and edits.

The authors apologize for these errors and state that this does not change the scientific conclusions of the article in any way. The original article has been updated.

